# Dilemmas and Challenges in the Anesthetic Management of Liver Transplantation for Transthyretin Amyloidosis in the Asian Context: A Case Report

**DOI:** 10.7759/cureus.82784

**Published:** 2025-04-22

**Authors:** Jacklyn Yek, Steffi Chan, Sook Muay Tay, Selene Tan

**Affiliations:** 1 Division of Anaesthesiology and Perioperative Medicine, Singapore General Hospital, Singapore, SGP

**Keywords:** ala117ser, anesthesia, arrhythmias, cardiac amyloidosis, living-related liver transplantation, pacing, transthyretin amyloidosis

## Abstract

Transthyretin (TTR) amyloidosis is a progressive, debilitating, and eventually fatal disease that is under-recognized and underdiagnosed in Asian patients. Liver transplantation is performed in patients with hereditary transthyretin amyloidosis to remove the source of abnormal transthyretin production to slow disease progression. There is uncertainty about the risk-benefit ratio due to the less favourable five-year survival of liver transplantation for Asian patients with non-Val30Met transthyretin amyloidosis, as the heterogeneity of non-Val30Met mutations and the potential for disease progression despite transplantation can make transplant outcomes unpredictable. Additionally, limited access and the prohibitive costs of disease-modifying therapies may influence the decision-making process with regard to liver transplantation.

We present a case of a 57-year-old male with Ala117Ser TTR amyloidosis and clinical manifestations of polyneuropathy, cardiac amyloidosis, and autonomic neuropathy. He received a living-related liver transplant. We describe the dilemmas and challenges in the anesthetic planning and management of this patient. Along with limited scientific data on liver transplantation for non-Val30Met TTR amyloidosis patients, we faced challenges in the management of intraoperative tachyarrhythmias, which eventually necessitated the use of a relatively contraindicated drug, verapamil, to achieve rate control. The lack of specific data meant that we had to base some decisions on practical wisdom.

## Introduction

Familial amyloid polyneuropathies (FAPs) are a group of hereditary, life-threatening multisystem disorders most commonly attributed to mutated transthyretin (TTR). While wild-type TTR amyloidosis also exists, it is a distinct, non-hereditary form of the disease [1]. The disease has devastating effects on patients and is characterized by varying degrees of progressive neuropathy and cardiomyopathy [2]. Other manifestations include autonomic neuropathy and renal fibrosis, with death occurring within a mean of 10 years from diagnosis [3].

Our case report describes an ethnic Chinese Singaporean patient who underwent LT for TTR amyloidosis. It highlights the key dilemmas in clinical decision-making, including the challenges in the use of intraoperative antiarrhythmic agents in the setting of cardiac amyloidosis and the management of perioperative arrhythmias with a background of cardiac amyloidosis. Through this case, we hope to shed light on the anaesthetic implications of liver transplantation in amyloidosis and stimulate further discussion on optimal perioperative strategies for these complex patients.

## Case presentation

**Figure 1:**
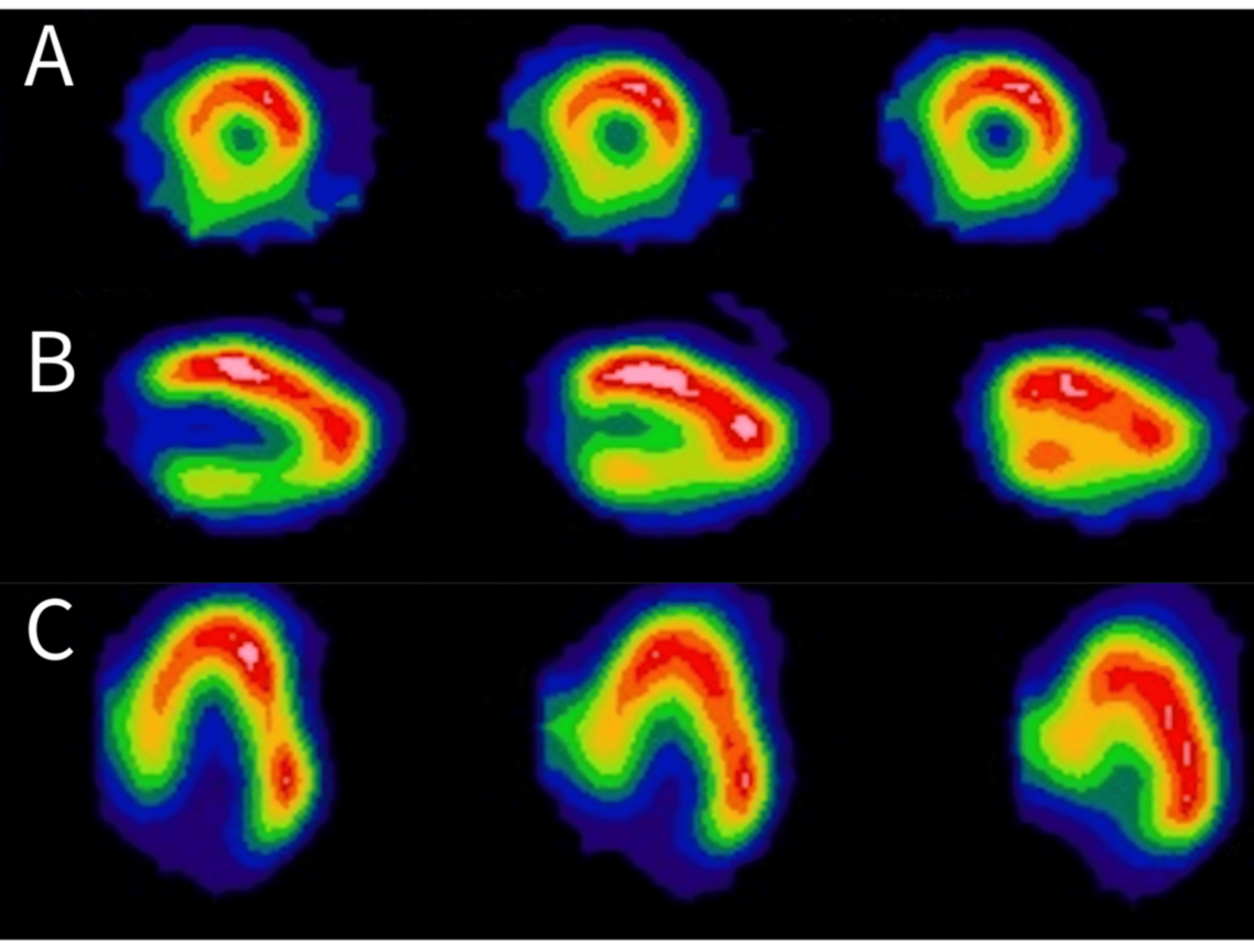
Positive Tc-99 m PYP scan demonstrating increased myocardial tracer uptake. Cardiac SPECT showing diffuse myocardial tracer uptake in short-axis images (A, top row), horizontal long-axis (B, middle row), and vertical-long axis images (C, last row). This scan is diagnostic of ATTR cardiomyopathy. Tc-99 m PYP: Technetium-99 m pyrophosphate; SPECT: single photon emission computed tomography; ATTR: transthyretin amyloid

A 57-year-old Chinese male (167 cm, 59.3 kg, BMI 21.2 kg/m2) with a past medical history of well-controlled hypertension and hereditary transthyretin (TTR) amyloidosis (Ala117Ser mutation) was referred to our hospital. His liver enzymes and bilirubin levels were normal. His initial clinical presentation consisted of glove-and-stocking polyneuropathy and gastrointestinal symptoms such as dysphagia, nausea, and vomiting. He was started on tafamidis, a selective stabilizer of TTR [4], after his diagnosis. After the initial diagnosis, he was diagnosed with cardiac amyloidosis through Technetium-99 m pyrophosphate (Tc-99 m PYP) scintigraphy, which demonstrated increased myocardial tracer uptake (Figure 1). The myocardial tracer uptake was greater than rib uptake, and the heart-to-contralateral lung ratio was 1.6, strongly suggestive of cardiac amyloidosis. He had no significant cardiac symptoms apart from orthostatic hypotension. Formal autonomic function testing was not performed, as the clinical features were suggestive of autonomic dysfunction. Given that anaesthetic management would be guided by the presumed diagnosis regardless of formal confirmation, further testing was not deemed necessary at this stage. An echocardiogram and 24-hour Holter monitoring test were performed, which did not demonstrate any arrhythmias. Due to the progression of his neuropathy despite pharmacological therapy, he was referred to the liver transplant team for consideration of a living-related liver transplant approximately two years after his diagnosis. His spouse was deemed suitable for liver donation, however, the surgery had to be delayed by another nine months to allow time for lifestyle optimization for the donor.

Due to the potential for drug-resistant bradyarrhythmias due to underlying cardiac amyloidosis, further compounded by the inherent fluid shifts of a liver transplant, temporary transvenous pacing wires (TPW) were electively inserted via the right internal jugular vein one day before surgery. This decision was made in consideration of the patient’s pre-existing comorbidities and the expected intraoperative hemodynamic challenges. Standard American Society of Anesthesiologists monitors, a bispectral index (BIS) monitor, and external defibrillation pads were placed prior to induction of anesthesia. The transvenous pacer was set at a backup rate of 60 beats per minute (bpm). Anesthesia was induced judiciously with 2 mg of midazolam and 80 mg of propofol, followed by sevoflurane maintenance between a minimum alveolar concentration between 0.6 and 0.8, titrated to a BIS of 40-60, titrated to the patient’s response. Induction agents were intentionally lowered to avoid large hemodynamic swings given his premorbid cardiac amyloidosis and autonomic dysfunction. In addition, invasive radial arterial blood pressure monitoring, left internal jugular vein central venous access and pressure monitoring, and insertion of a transesophageal echocardiography (TEE) probe were performed before skin incision. The following drug infusions were administered during the surgery: fentanyl 150-200 mcg/hour, calcium chloride infusion titrated to ionized calcium readings on arterial blood gas monitoring, and atracurium infusion 30 mg/hr to maintain adequate muscle relaxation for surgery. Flotrac™ (Edwards Lifesciences, Irvine, CA, US) monitoring (cardiac output, cardiac index, systemic vascular resistance, and stroke volume variation) was used for goal-directed fluid therapy in addition to TEE for cardiac monitoring (Video 1).

**Video 1:** Patient's TEE findings of thickened left ventricular walls and significant diastolic dysfunction, suggestive of cardiac amyloidosis TEE: transesophageal echocardiography

The administration of blood products was guided both clinically and with ROTEM^®^ thromboelastography. Prophylactic antibiotics were given perioperatively and regularly.

Liver explantation proceeded uneventfully. However, during the anhepatic phase, the patient developed narrow complex supraventricular tachycardia (SVT) between 150 to 170 bpm with no immediate hemodynamic compromise. Intravenous (IV) adenosine 6 mg was administered, with a transient return to sinus rhythm. Two further doses of IV adenosine (12 mg) were administered, which successfully restored sinus rhythm. Amiodarone was not administered due to concerns regarding drug-induced hepatotoxicity [5], particularly in consideration of preserving the partial graft function in the context of a living-related liver transplant. Thirty mmol of intravenous magnesium sulfate was administered to target a magnesium level of 1.0 mmol/L. The arterial blood gas result at this point was: pH 7.282, base excess -13 mmol/L, potassium 3.1 mmol/L, and lactate 8.87 mmol/L. Intravenous solutions of 20 mmol of potassium chloride and 100 mmol of sodium bicarbonate were administered.

During inferior vena cava cross-clamping for the hepatic vein and portal vein anastomosis, the patient developed severe hypotension, likely due to the underlying diastolic dysfunction, which required support with noradrenaline (up to 0.3 mcg/kg/min) and adrenaline infusions (up to 0.1 mcg/kg/min). The hypotensive episodes were challenging to manage, often only partially responsive to treatment with vasopressors, inotropes, and fluids, resulting in transient but significant periods where the MAP was below 60 mmHg.

After an anhepatic time of 60 minutes, reperfusion was commenced. During the reperfusion phase, he developed atrial fibrillation with a rapid ventricular response of between 150 to 160 bpm, for which IV esmolol was given in aliquots of 10 mg (total 40 mg). A short-acting beta blocker like esmolol was used in this instance for rate control to improve cardiac output. Blood pressure was maintained with noradrenaline between 0.2 to 0.3 mcg/kg/min. In view of the limited response to esmolol, IV verapamil 1 mg was administered, which resulted in sustained rate control and allowed conversion to sinus rhythm. The rest of the surgery was completed uneventfully. The actual graft weight was slightly small-for-size, weighing 399 g with a calculated graft-to-recipient weight ratio of 0.67. Hemoglobin was 8 g/dL, and lactate at the end of surgery was 13 mmol/L, which was elevated due to the effects of hypotension from an ischemic-reperfusion injury.

The patient was transferred intubated and sedated to the surgical intensive care unit (SICU) in a relatively stable state on noradrenaline and adrenaline infusions (between 0.05 and 0.07 mcg/kg/min), which were weaned off shortly after transfer to the SICU. The Flotrac monitor was kept on to trend the cardiac output and guide fluid therapy. He was extubated uneventfully 12 hours postoperatively. Routine hepatic US Doppler was normal, and lactate levels normalized by postoperative day two, demonstrating good graft function. No further septic screening was performed, as clinical and laboratory findings were not suggestive of sepsis. 

The patient remained well until postoperative day 7, when he developed fulminant neutropenic septicaemia secondary to Escherichia coli, likely of abdominal origin. The patient experienced further rapid deterioration and eventually passed away from septic shock. No further salvage interventions were considered due to the extent of multiorgan failure.

## Discussion

Contrary to initial beliefs that transthyretin-related familial amyloid polyneuropathy was endemic to certain areas, it is now known to occur worldwide [2]. More than 100 point mutations of the TTR gene with varying disease presentations and geographic locations have been described, the most common being the Val30Met(V30M) mutation, which is endemic to Portugal, Sweden, and Japan [3]. There are considerably fewer studies on transthyretin amyloidosis in the Asian population in the literature. A study of 29 patients in a multiracial Southeast Asian cohort over a 13-year period identified ATTR-A97S (Ala117Ser) as the most common variant, constituting 66.7% of ethnic Chinese patients and 48.3% of the cohort. The most common presentations were somatic neuropathy, carpal tunnel syndrome, autonomic dysfunction, and cardiac dysfunction [1]. TTR is a protein mainly produced in the liver, and liver transplantation (LT) for the treatment of TTR amyloidosis has shown favorable results for FAP patients with the V30M mutation, with a five-year survival rate of 100% [4]. However, the applicability of studies on primarily Western populations with the V30M mutation to the Southeast Asian population with non-V30M is questionable. LT performed in non-TTR V30M cases is associated with a lower five-year survival rate [6]. TTR amyloidosis in the Asian population is not well-recognized and may be underdiagnosed due to limited access to healthcare, availability of specific tests, and expertise in assessing diagnostic images [7,8]. The impact of TTR amyloidosis in Asia is likely to be more significant and far-reaching than what current scientific literature suggests.

Several dilemmas and challenges are highlighted in this case report. The risk-benefit ratio of LT for Asian patients with hereditary non-V30M TTR amyloidosis is less certain. While the five-year survival rate for liver transplantation in patients with the V30M mutation is well-studied, there is a paucity of equivalent data on patients with other forms of TTR amyloidosis such as the Ala117Ser mutation that our patient carried. Reports have demonstrated a progression of cardiomyopathy and neuropathy in numerous patients with non-V30M TTR mutations despite liver transplantation [9,10]. Liver transplant surgery is a technically challenging procedure with significant risks of mortality and postoperative complications such as infection and organ rejection. It involves replacing an otherwise healthy liver to eliminate the source of TTR formation and slow down disease progression. New therapies may possibly replace liver transplant as the gold standard for the treatment of TTR amyloidosis, for which tafamidis is the current approved and primary treatment therapy [11,12].

There are various emerging disease-specific treatments, such as TTR tetramer stabilizers (tafamidis, acoramidis), silencers of the gene encoding TTR (Patisiran, Inotersen, Eplontersen, CRISPR-Cas9 gene editing therapy), and antibodies (destruction and reabsorption of formed amyloid tissue deposits) [12]. Unfortunately, depending on the region, access to disease-modifying drugs may be limited due to prohibitive costs. Due to their high costs experienced worldwide and classification as non-standard drugs, which limits access to insurance claims, patients are sometimes prescribed only symptomatic treatment [11,12]. Tafamidis has a list price of approximately $268,000 per year, and an estimated 92.6% price reduction from $225,000 to $16563 would be necessary to make Tafamidis cost-effective at $100,000/quality-adjusted life-year.

The next challenge would be weighing the risk-benefit ratio of preoperative pacemaker implantation for patients with TTR cardiac amyloidosis. The main cause of perioperative death in LT is related to cardiac difficulties in 39% of patients [2]. Progressive heart failure and fatal cardiac arrhythmias in the perioperative period have been reported extensively [13]. Literature suggests that a pacemaker is advisable in patients with a history of dizziness, syncope, Holter ECG changes of sinoatrial or atrioventricular heart block, autonomic circulatory disturbances [14] as well as existing conduction disturbances [15]; however, this practice varies based on physician discretion. Current evidence does not support prophylactic pacemaker insertion in cardiac amyloidosis patients undergoing LT [15]. In addition, while pacemaker implantation can help provide symptomatic relief, it does not confer a mortality benefit [16].

Although our patient did not have inducible high-grade AV conduction defects, pacing wires were pre-emptively placed due to the well-documented post-reperfusion syndrome, where electrolyte and pH disturbances during the anhepatic and neohepatic phases could precipitate malignant arrhythmias. This decision was made based on our institutional experience. Cardiac amyloidosis is known to be very resistant to cardiosupportive treatment [17,18], which could explain why the hypotension experienced intraoperatively was only partially responsive to therapy, due to underlying diastolic dysfunction and autonomic dysfunction. Patients with pre-existing autonomic neuropathy may have atropine-resistant bradyarrhythmias, which may necessitate emergency cardiac pacing [17,18].

The choice of drug in the management of arrhythmias is also of concern. Acute amiodarone-induced hepatotoxicity after LT has previously been described, with an incidence of 24-26% [7]. The omission of amiodarone was a case-specific decision. The presentation of amiodarone-induced hepatotoxicity mimics the more frequent causes of post-LT hepatic dysfunction, including ischemic-reperfusion hepatic injury, hepatic artery thrombosis, rejection, or biliary obstruction, and could hence lead to diagnostic confusion. In Asia, living-related liver transplants are performed in higher numbers due to low deceased organ donor rates [19]. We opted not to use amiodarone in view of the slightly small-for-size graft. Commonly used drugs, such as beta-blockers, calcium-channel blockers, and digoxin, are also known to be poorly tolerated in TTR cardiac amyloidosis patients due to the restrictive filling physiology and heart rate dependence, favoring the adoption of the rhythm control strategy. In this case, a short-acting beta-blocker (esmolol) was trialed to assess its hemodynamic tolerability given its transient effect, before considering the use of longer-acting beta-blockers.

Drugs like digoxin and verapamil may bind to amyloid fibrils and cause a heart block [20]. Our patient developed atrial fibrillation with rapid ventricular response during surgery. Verapamil was chosen over diltiazem, as it was more cardioselective. Although verapamil is relatively contraindicated in patients with cardiac amyloidosis [20], it was the only agent that achieved sustained rate control for our patient. The patient did not develop heart failure or hypotension in response to IV verapamil 1 mg.

Unfortunately, the patient emerged from surgery in a stable condition but succumbed one week later to postoperative septicemia despite antibiotics prophylaxis, a known cause of mortality after liver transplantation for amyloidosis.

## Conclusions

This case report highlights the unique anesthetic challenges encountered during liver transplantation in an Asian patient with hereditary transthyretin amyloidosis carrying a non-Val30Met mutation. The coexistence of cardiac amyloidosis and the need to preserve graft function in a living-related transplant presented significant dilemmas in selecting appropriate intraoperative antiarrhythmic therapies. This case underscores the importance of individualized anesthetic planning, careful risk-benefit assessment, and close interdisciplinary coordination in the perioperative management of patients with systemic amyloidosis undergoing major surgery.
